# Acute Effects of Positive Airway Pressure on Functional Mitral Regurgitation in Patients with Systolic Heart Failure

**DOI:** 10.3389/fphys.2017.00921

**Published:** 2017-11-23

**Authors:** Takao Kato, Takatoshi Kasai, Shoichiro Yatsu, Azusa Murata, Hiroki Matsumoto, Shoko Suda, Masaru Hiki, Nanako Shiroshita, Mitsue Kato, Fusae Kawana, Sakiko Miyazaki, Hiroyuki Daida

**Affiliations:** ^1^Department of Cardiovascular Medicine, Juntendo University School of Medicine, Tokyo, Japan; ^2^Cardiovascular Respiratory Sleep Medicine, Juntendo University Graduate School of Medicine, Tokyo, Japan

**Keywords:** adaptive servo-ventilation, continuous positive airway pressure, cardiac output, congestion, filling pressure

## Abstract

**Background:** Acute effects of positive airway pressure (PAP) [including continuous PAP (CPAP) and adaptive servo-ventilation, an advanced form of bi-level PAP] on functional mitral regurgitation (fMR) in patients with heart failure (HF) with left ventricular (LV) systolic dysfunction remain unclear. Thus, whether PAP therapy reduces fMR in such patients with HF was investigated.

**Methods and Results:** Twenty patients with HF and LV systolic dysfunction defined as LV ejection fraction (LVEF) <50% (14 men; mean LVEF, 35.0 ± 11.5%) with fMR underwent echocardiography during 10-min CPAP (4 and 8 cm H_2_O) and adaptive servo-ventilation. For fMR assessment, MR jet area fraction, defined as the ratio of MR jet on color Doppler to the left atrial area, was measured. The forward stroke volume (SV) index (fSVI) was calculated from the time-velocity integral, cross-sectional area of the aortic annulus, and body surface area. fMR significantly reduced on CPAP at 8 cm H_2_O (0.30 ± 0.12) and adaptive servo-ventilation (0.29 ± 0.12), compared with the baseline phase (0.37 ± 0.12) and CPAP at 4 cm H_2_O (0.34 ± 0.12) (*P* < 0.001). The fSVI did not change in any of the PAP sessions (*P* = 0.888). However, significant differences in fSVI responses to PAP were found between sexes (P for interaction, 0.006), with a significant reduction in fSVI in women (*P* = 0.041) and between patients with baseline fSVI ≥ and < the median value (27.8 ml/m^2^, P for interaction, 0.018), with a significant fSVI reduction in patients with high baseline fSVI (*P* = 0.028). In addition, significant differences were found in fSVI responses to PAP between patients with LV end-systolic volume (LVESV) index ≥ and < the median value (62.0 ml/m^2^, P for interaction, 0.034), with a significant fSVI increase in patients with a high LVESV index (*P* = 0.023).

**Conclusion:** In patients with HF, LV systolic dysfunction, and fMR, PAP can alleviate fMR without any overall changes in forward SV. However, MR alleviation due to PAP might be associated with a decrease in forward SV in women with high baseline SV, whereas MR alleviation due to PAP might be accompanied by increased forward SV in patients with a dilated LV.

## Introduction

Positive airway pressure (PAP) delivered by face masks has been used in the wide spectrum of heart failure (HF) care (Bradley et al., 2005; Arzt et al., 2007; Wang et al., 2007; Gray et al., 2008; Kasai et al., 2008; Kato et al., 2014; Khayat et al., 2015; Momomura et al., 2015; Nakano et al., 2015; Kinoshita et al., 2017). For instance, in patients with acute decompensated HF, continuous PAP (CPAP) resulted in early physiologic improvements and reduced the need for intubation and mechanical ventilation, indicating that PAP has beneficial hemodynamic and respiratory effects (Bersten et al., 1991). Several studies have suggested that bi-level PAP, which provides fixed pressure support (PS) during inspiration in addition to expiratory PAP (EPAP) and backup ventilation with a preset respiratory rate, is also an effective mode of PAP in patients with acute decompensated HF (Hoffmann and Welte, 1999; Rusterholtz et al., 1999; Masip et al., 2000). More recently, adaptive servo-ventilation (ASV), which is an advanced mode of bi-level PAP and automatically provides altering PS for each inspiration with an EPAP in addition to backup ventilation with variable respiratory rates, leading to stabilization of respiration (see Figure S1), was reported to be useful for patients with acute decompensated HF (Nakano et al., 2015; Kinoshita et al., 2017).

In contrast, patients with chronic stable HF had inconsistent acute hemodynamic responses to PAP, which were possibly associated with differences in the type of PAP and the patients' profiles and conditions (Bradley et al., 1992; De Hoyos et al., 1995; Liston et al., 1995; Kiely et al., 1998; Philip-Joet et al., 1999; Mehta et al., 2000; Steiner et al., 2008; Haruki et al., 2011; Yoshida et al., 2012; Yamada et al., 2013). CPAP increases stroke volume (SV) in patients with HF with a high LV filling pressure and/or LV chamber size (Bradley et al., 1992; De Hoyos et al., 1995; Steiner et al., 2008; Yoshida et al., 2012). Similar responses in those patient populations were observed when bi-level PAP or ASV was applied (Philip-Joet et al., 1999; Yamada et al., 2013). Recently, adding PS on CPAP was reported to more favorably affect SV than isolated CPAP, possibly through lung inflation and its induced reflex inhibition of sympathetic nerve activity (Yoshida et al., 2012). In addition, stabilization of respiration by altering PS and servo-control ventilation of ASV was suggested to play important roles in sympathoinhibitory effects (Ushijima et al., 2014).

In patients with chronic HF, PAP has been used to alleviate sleep-disordered breathing (SDB), which is a frequent complication known to be associated with worse clinical outcomes (Kasai and Bradley, 2011; Kasai, 2012; Kasai et al., 2012). In patients with HF and SDB, PAP resulted in improvements in cardiac function in many small-scale and relatively short-term randomized controlled trials (Kasai and Bradley, 2011; Kasai, 2012; Kasai et al., 2012; Kato et al., 2014); however, two clinical trials that investigated the effects of SDB treatment with PAP on long-term clinical outcomes in patients with chronic HF failed to show any benefit of PAP against mortality and/or adverse clinical events (Bradley et al., 2005; Cowie et al., 2015). Even in those studies, PAP therapy might be beneficial in specific subgroups of patients, such as those with central sleep apnea, which can be alleviated by CPAP, those with less Cheyne–Stokes respiration, and those with less impaired left ventricular (LV) ejection fraction (LVEF) (Arzt et al., 2007; Cowie et al., 2015). Considering these facts, there might be subgroups of patients with HF with specific profiles or conditions who are responsive to PAP therapy. These profiles or conditions may include functional mitral regurgitation (fMR).

fMR, arising from mitral annular dilation and/or papillary muscle dysfunction in association with LV dysfunction, is a common finding in patients with HF (Asgar et al., 2015). It contributes to the elevation of LV end-diastolic pressure as well as left and right atrial pressure and causes progressive dilatation of the LV (Vanoverschelde et al., 1990), leading to a vicious cycle. However, the presence of significant fMR suggests that intensive treatment would be beneficial (Keren et al., 1989; Evangelista-Masip et al., 1992). Indeed, short-term application of CPAP or bi-level PAP was reported to alleviate fMR rapidly in patients with acute decompensated HF (Bellone et al., 2004). In contrast, short-term application of ASV did not rapidly alleviate fMR in a study on patients with stable HF (Haruki et al., 2011). In any case, inconsistent acute responses of fMR to PAP might possibly be associated with differences in the type of PAP and patients' profiles and conditions.

The aim of our study was to evaluate the acute effect of several settings of PAP therapy on fMR, taking into account changes in other hemodynamic parameters. Our specific hypothesis is that fMR will be alleviated because an increased CPAP level and ASV have similar effects and that alleviation of fMR will be accompanied by changes in SV, which will be more prominent in certain subgroups of patients.

## Materials and methods

### Subjects

We enrolled patients with systolic HF at Juntendo University Hospital (Tokyo, Japan) if they met following criteria: (1) men and women aged ≥20 years, (2) HF due to ischemic or non-ischemic cardiomyopathy, (3) LVEF <50% on echocardiography, (4) mild to severe fMR, (5) stable clinical status evidenced by the absence of acute exacerbations of dyspnea, and (6) underwent overnight polysomnography to assess for SDB. The exclusion criteria were (1) patients with shock, (2) those requiring catecholamine and/or inotrope infusion, (3) those requiring mechanical ventilation or supplemental oxygen, (4) those with organic MR, (5) those with moderate to severe aortic regurgitation and/or stenosis, (6) those with a history of chronic lung disease, and (7) those with dialysis.

The subjects' characteristics and medications at the time of this study were recorded. The estimated glomerular filtration rate (Matsuo et al., 2009) and B-type natriuretic peptide levels were assessed in the early morning of the day of the experiment. All patients underwent overnight polysomnography on the night before the experiment. Apneas and hypopneas were quantified, and the severity of SDB was assessed using the frequency of apneas and hypopneas per hour of sleep (i.e., apnea–hypopnea index, AHI). Patients were divided into an obstructive-dominant group (≥50% had obstructive apnea) and a central-dominant group (>50% had central apnea).

This study was approved by the Juntendo University Hospital Institutional Review Board, and the study complied with the ethical principles of the Declaration of Helsinki. Written informed consent was obtained from all participants.

### Echocardiography

Standard two-dimensional echocardiography and Doppler ultrasound examinations were performed using a commercially available ultrasound imaging system (Vivid I, GE Healthcare, Milwaukee, WI, USA). Left ventricular end-diastolic volume (LVEDV), LV end-systolic volume (LVESV), stroke volume (SV), and LVEF were calculated using Simpson's biplane method. LVEDV, LVESV, and SV were divided by the body surface area and expressed as the LVEDV, LVESV, and SV indexes. The degree of MR was assessed from 0 (none) to 4 (severe) and was based on the MR jet area fraction, which was defined as the ratio of MR jet area to the left atrial (LA) area. Because SV determined using Simpson's method may consist of both forward and MR flows (Giannuzzi et al., 1991), forward SV was also calculated as the LV outflow tract area × velocity time integral of the LV outflow velocity. Systemic vascular resistance (SVR) was calculated as (mean BP − central vein pressure [CVP]/[forward SV × HR]) × 80. CVP was estimated by the diameter of the inferior vena cava (IVC), and by the presence or absence and degree of the respiratory variation of the IVC (Lanzarini et al., 2002). Forward SV and SVR were also divided by the body surface area and expressed as indices. Diastolic function was evaluated using transmitral flow velocity and average e′ from the septal and lateral tissue Doppler samplings (Nagueh et al., 2009). All images were recorded by one investigator in at least three cardiac cycles and were assessed by another investigator who was blinded to the clinical data and settings of PAP. The final values represented the mean of at least three measurements for patients with regular rhythm and five measurements for those with atrial fibrillation (AF).

Intra-observer variability was determined by having one observer repeat the measurement of MR jet area fraction and forward SV in 10 randomly selected patients three times. Repeated measurements were performed 1 week apart. Inter-observer variability was determined by having a second observer measure these variables in the same datasets. Intra- and inter-observer variabilities were assessed by values calculated as the standard deviation of the corresponding three or two measurements as a percentage of the mean.

### PAP and study protocol

All PAPs were applied with a full-face mask, using the BiPAP Auto-SV Advanced system (Philips Respironics, Murrysville, PA, USA), with the patients awake. After BP and HR were stabilized, the baseline step was started without PAP for 10 min, during which baseline echocardiography was performed. BP and HR were measured twice on the right upper arm in each step, using an automated vital signs monitoring device (DASH3000, GE Health Care, UK Ltd., Milwaukee, WI, USA). Subsequently, CPAP at 4 cm H_2_O, 8 cm H_2_O, and ASV with an EPAP of 4 cm H_2_O, an automated PS ranging from 0 to 4 cm H_2_O, and an automated backup ventilation mode (Figure S1) were applied for 10 min in a random order. In each 10-min period, repeated echocardiography was performed. After these three steps with PAP, all measurements were repeated without PAP (i.e., post step).

### Statistical analysis

In this study, values are expressed as mean ± standard deviation or median (interquartile range) for continuous variables and numbers (%) for nominal variables. Variations in echocardiography parameters across each step were assessed with one way repeated-measures analysis of variance (ANOVA) and the Tukey test for multiple comparisons. To assess the potential heterogeneity of the effect of PAP on fMR, forward SV index (fSVI), and SVR index, we performed subgroup analyses as exploratory analyses. The subgroups included sex, age (cutoff, median of 71 years), body mass index ([BMI], cutoff, median of 21.7 kg/m^2^), presence or absence of AF, type of predominant SDB, BNP level (cutoff, median of 415.8 pg/ml), right ventricular systolic pressure (RVSP) by echocardiography (cutoff, median of 34.2 mmHg), baseline LVEDV index (cutoff, median of 91.2 ml/m^2^), baseline LVESV index (cutoff, median of 62.0 ml/m^2^), baseline fSVI (cutoff, median of 27.8 ml/m^2^), and baseline MR degree. The first-order interactions in two-way repeated measures ANOVA models for fMR, forward SV and SVR were examined by entering interaction terms between PAP steps (i.e., baseline, CPAP at 4 cm H_2_O, 8 cm H_2_O, ASV, and post steps) and the above-mentioned subgroup (i.e., step-by-subgroup interaction). If significant step-by-subgroup interactions were found, one-way repeated-measures ANOVA was performed within each subgroup. A *P* < 0.05 indicated statistical significance. Analyses were performed using SPSS ver. 23.0 (IBM Corp., Armonk, NY, USA).

## Results

### Baseline patient characteristics

Overall, 23 eligible patients were enrolled in this study. However, three were excluded because of technical difficulty while measuring echocardiography parameters; thus, the data of 20 patients were assessed. The characteristics of these patients, including 14 men and 6 women, are summarized in Table 1. Most of them were elderly, non-obese, and had HF with New York Heart Association class II symptoms. Half of them had ischemic etiology, and three had AF. Of the five patients with pacemaker, none were pacemaker dependent during the study period. All of them had an AHI ≥ 5 events/h of sleep, and seven of them were in the central-dominant group.

**Table 1:** Patient characteristics.

***n* = 20**	
Age, years	67.6 ± 14.3
Female sex, *n* (%)	6 (30)
Body mass index, kg/m^2^	22.1 ± 4.1
NYHA class—I, *n* (%)	4 (20)
II, *n* (%)	11 (55)
III, *n* (%)	5 (25)
Ischemic etiology, *n* (%)	10 (50)
Pacemaker, *n* (%)	5 (25)
CRT, *n* (%)	2 (10)
Atrial fibrillation, *n* (%)	3 (15)
eGFR, ml/min/1.73 m^2^	52.4 ± 21.9
Plasma BNP, pg/ml	415.8 (589.9)
AHI, events/h of sleep	28.8 ± 16.5
AHI ≥ 5 event/h of sleep, *n* (%)	20 (100)
Central-dominant group, *n* (%)	7 (35)
**MEDICATIONS**	
Diuretics, *n* (%)	17 (85)
Aldosterone blockers, *n* (%)	13 (65)
ACE-Is/ARBs, *n* (%)	18 (90)
Beta blockers, *n* (%)	17 (85)

*Values are expressed as mean ± standard deviation or median (interquartile range) for continuous variables and numbers (%) for nominal variables. ACE, angiotensin-converting enzyme; AHI, apnea-hypopnea index; ARB, angiotensin II receptor blockers; BNP, B-type natriuretic peptide; CRT, cardiac resynchronization therapy; eGFR, estimated glomerular filtration rate; NYHA, New York Heart Association*.

Echocardiography data are shown in Table 2. The patients had severely impaired LVEF and dilated LV chamber sizes, which were associated with a high LV filling pressure. None of the subjects had significant adverse effects due to PAP application, and all of them completed the PAP trial. During ASV mode, the mean applied PS was 1.0 ± 0.4 cm H_2_O above EPAP of 4 cm H_2_O.

**Table 2:** Baseline echocardiography parameters.

***n* = 20**	
IVST, mm	9.6 ± 2.0
PWT, mm	9.3 ± 2.0
LVEDV index, ml/m^2^	90.3 ± 30.1
LVESV index, ml/m^2^	60.2 ± 25.1
LVEF, %	35.0 ± 11.5
SV index, ml/m^2^	30.1 ± 9.8
Forward SV index, ml/m^2^	27.5 ± 9.7
SVR index, dyne*s/cm^5^/m^2^	1,592 ± 593
E, m/s	82.5 (34.5)
A, m/s^*^	39.0 (21.0)
DcT, ms	163.4 (77.0)
e′ mid, m/s	4.0 (2.0)
E/e′	20.5 (8.5)
**Degree of MR**, ***n*** **(%)**
Mild	5 (25)
Moderate	10 (50)
Severe	5 (25)
ERO area, cm^2^	0.31 ± 0.09
RVol, ml	44.8 ± 14.3
MR jet area fraction	0.37 ± 0.12
IVC, mm	17.0 ± 6.4
RVSP, mmHg	35.7 ± 18.0

*Values are expressed as mean ± standard deviation or median (interquartile range) for continuous variables and numbers (%) for nominal variables*.

* *n = 17 due to atrial fibrillation. Dct, deceleration time; ERO, effective regurgitant orifice; IVC, inferior vena cava; IVST, intraventricular septal thickness; LVEDV, left ventricular end-diastolic volume; LVEF, left ventricular ejection fraction; LVESV, left ventricular end-systolic volume; MR, mitral regurgitation; PWT, posterior wall thickness; RVol, regurgitant volume; RVSP, right ventricular systolic pressure; SV, stroke volume; SVR, systemic vascular resistance*.

### Effects of PAP on BP, HR, and echocardiography parameters

Intra-observer variabilities for measuring MR jet area fraction and fSVI were 4.8 and 5.0%, respectively. Corresponding inter-observer variabilities were 7.9 and 8.7%, respectively.

**Figure 1:**
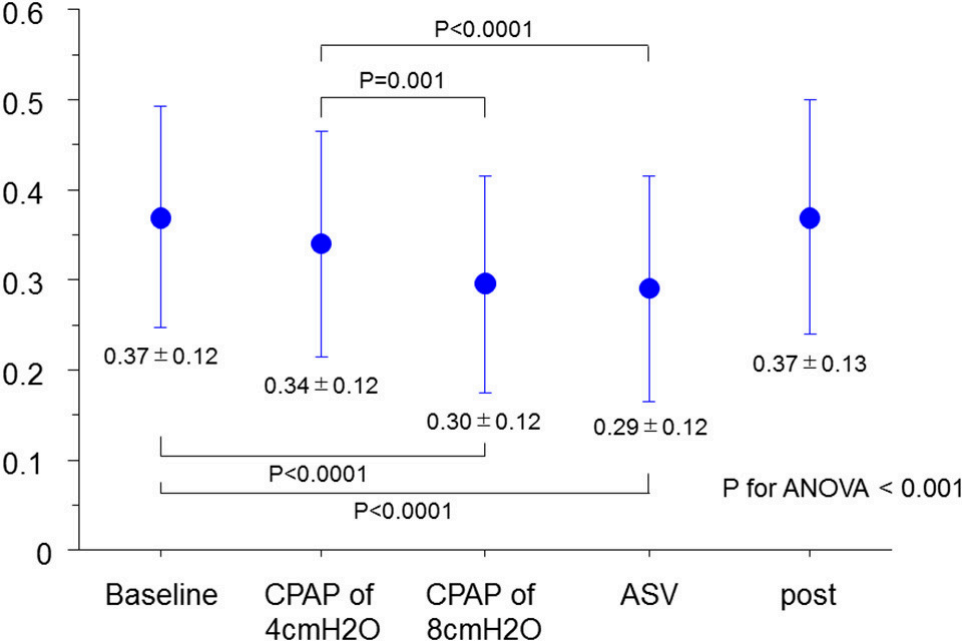
Changes in MR jet area fraction. ANOVA, analysis of variance; ASV, adaptive-servo ventilation; CPAP, continuous positive airway pressure; MR, mitral regurgitation.

**Figure 2:**
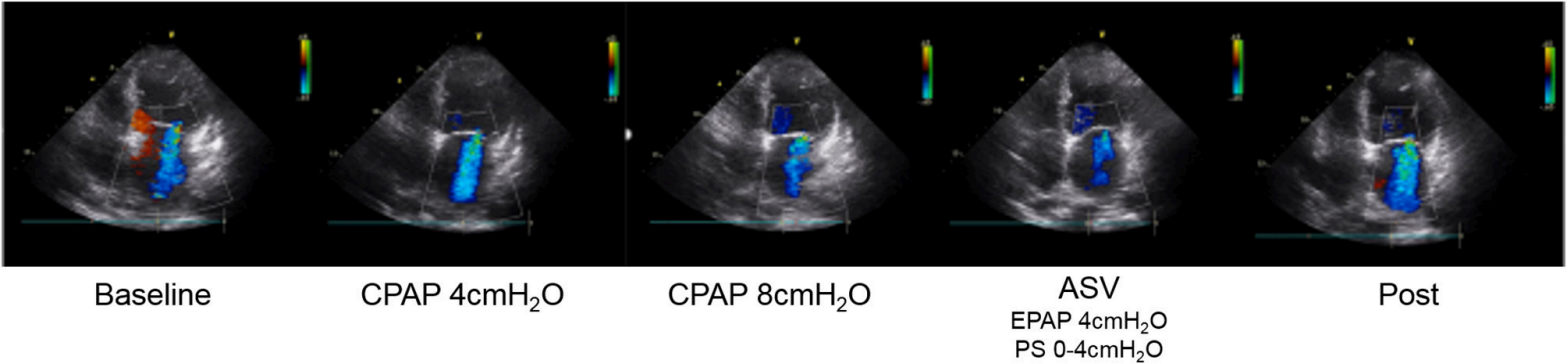
Representative color Doppler images of functional MR. MR jet area fraction reduced due to PAP therapy, especially CPAP at 8 cm H_2_O and ASV. ASV, adaptive-servo ventilation; CPAP, continuous positive airway pressure; EPAP, expiratory positive airway pressure; MR, mitral regurgitation; PAP, positive airway pressure; PS, pressure support.

As shown in Figure 1, the MR jet area fraction was significantly reduced with the use of CPAP at 8 cm H_2_O and ASV compared with that at baseline and CPAP at 4 cm H_2_O. A dose-effect relationship was found across CPAP pressures from 0 (baseline) and 4–8 cm H_2_O, although no difference was observed in the reduction of MR jet area fraction between CPAP at 8 cm H_2_O and ASV. Representative color Doppler images for fMR are shown in Figure 2 (also see Supplementary Video). The fSVI did not change across the steps (Table 3). The changes in BP, HR, and other echocardiography data are summarized in Table 3. Diastolic BP significantly decreased in ASV compared to CPAP at 8 cm H_2_O. HR significantly decreased in CPAP at 4 cm H_2_O compared to baseline. However, no significant variations were found in the other parameters, although they tended to increase the diameter of the IVC.

**Table 3:** Changes in BP, HR, and echocardiographic data other than MR jet area fraction.

***N* = 20**	**Baseline**	**CPAP at 4 cm H_2_O**	**CPAP at 8 cm H_2_O**	**ASV**	**Post**	***P* for ANOVA**
Systolic BP, mmHg	104.0 ± 15.6	103.2 ± 15.1	106.1 ± 16.7	105.5 ± 15.5	105.3 ± 16.0	0.634
Diastolic BP, mmHg	63.7 ± 13.7	63.1 ± 12.3	65.8 ± 13.5	61.8 ± 11.0^*^	63.1 ± 11.5	0.049
HR, /min	67.6 ± 12.7	63.0 ± 10.9^†^	64.6 ± 12.6	63.8 ± 13.6	69.2 ± 12.5	0.031
LVEDV index, ml/m^2^	90.3 ± 30.1	91.0 ± 26.2	87.7 ± 26.3	89.2 ± 27.9	90.9 ± 28.0	0.591
LVESV index, ml/m^2^	60.2 ± 25.1	59.0 ± 20.7	56.3 ± 21.3	58.2 ± 23.5	59.6 ± 24.5	0.247
LVEF, %	35.0 ± 11.5	35.9 ± 10.0	35.4 ± 10.5	36.4 ± 7.7	35.3 ± 10.5	0.784
SV index, ml/m^2^	30.1 ± 9.8	32.0 ± 10.0	31.4 ± 9.5	31.0 ± 7.7	31.3 ± 7.8	0.834
Forward SV index, ml/m^2^	27.5 ± 9.7	27.7 ± 7.9	26.7 ± 7.5	27.5 ± 8.4	27.1 ± 8.0	0.888
SVR index, dyne*s/cm^5^/m^2^	1,592 ± 593	1,522 ± 483	1,574 ± 532	1,519 ± 554	1,467 ± 438	0.396
E/e'	20.5 (8.5)	21.0 (12.1)	21.3 (12.3)	19.1 (13.0)	18.0 (13.9)	0.324
IVC, mm	17.0 ± 6.4	17.1 ± 6.2	17.8 ± 5.8	17.5 ± 6.1	16.9 ± 6.5	0.085
RVSP, mmHg	35.7 ± 18.0	35.8 ± 17.5	36.0 ± 17.5	35.2 ± 18.4	38.2 ± 18.1	0.171

*Values are expressed as mean ± standard deviation or median (interquartile range)*.

* *P < 0.05 vs. CPAP at 8 cm H_2_O*.

† *P < 0.05 vs. baseline. ANOVA, analysis of variance; ASV, adaptive servo-ventilation; BP, blood pressure; CPAP, continuous positive airway pressure; HR, heart rate; IVC, inferior vena cava LVEDV, left ventricular end-diastolic volume; LVEF, left ventricular ejection fraction; LVESV, left ventricular end-systolic volume; MR, mitral regurgitation; RVSP, right ventricular systolic pressure; SV, stroke volume; SVR, systemic vascular resistance*.

### Subgroup analyses

**Figure 3:**
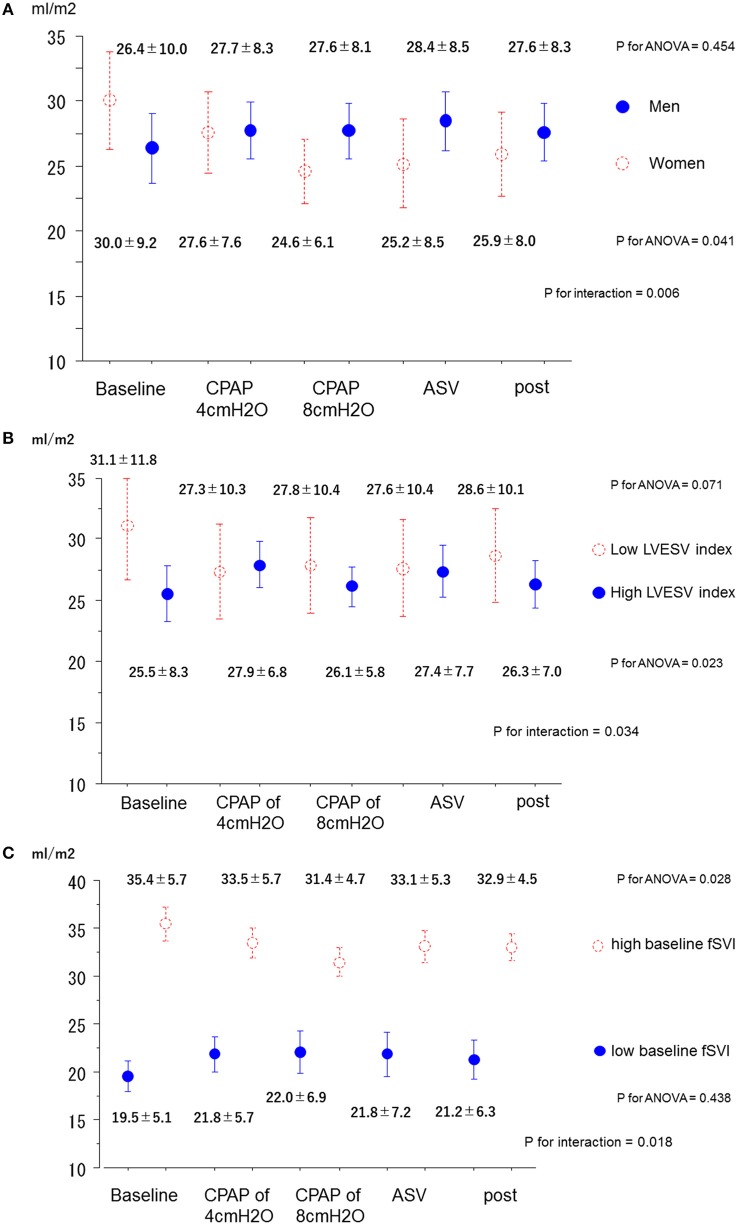
Changes in the forward SV index in the subgroups. **(A)** Men and women. **(B)** High and low LVESV index. **(C)** High and low baseline fSVI. ANOVA, analysis of variance; ASV, adaptive-servo ventilation; CPAP, continuous positive airway pressure; fSVI, forward stroke volume index; LVESV, left ventricular end-systolic volume.

No interactions were found between each subgroup and variation of MR jet area fraction. On the contrary, although no step-by-subgroup interactions were found between the variation of the fSVI and the subgroups of age, BMI, AF, type of predominant SDB, BNP, baseline RVSP, baseline LVEDV index, and baseline MR degree, significant differences were found in the variations of fSVI based on sex, baseline LVESV index, and baseline fSVI (P for interaction = 0.006, 0.034, and 0.017, respectively; Figures 3A–C). In men, the fSVI remained stable (*P* = 0.454), whereas in women, the fSVI decreased (*P* = 0.041) as the applied PAP levels increased. In addition, in patients with a low LVESV index, the fSVI tended to decrease (*P* = 0.071), whereas in those with a high LVESV index, fSVI significantly increased (*P* = 0.023). In patients with a low fSVI at baseline, fSVI remained stable (*P* = 0.438), whereas in those with a high fSVI at baseline, fSVI significantly decreased (*P* = 0.028). In terms of SVR index, although no step-by-subgroup interactions were found between variations of the SVR index and the subgroups of BMI, AF, central-dominant, BNP, baseline RVSP, baseline LVEDV index, baseline LVESV index, and baseline MR degree, a significant difference was found in the variations of the SVR index based on the baseline fSVI (P for interaction = 0.003), and sex and age subgroups tended to have different variations of the SVR index (P for interaction = 0.051, and 0.087, respectively; Figures S2A–C). Patients with a low fSVI at baseline showed a significant reduction in the SVR index as the applied PAP level increased (*P* < 0.001), whereas those with a high fSVI at baseline showed no change in the SVR index (*P* = 0.204).

## Discussion

This study has several important findings that provide insight into the alleviation of fMR due to PAP in patients with HF and LV systolic dysfunction. First, the degree of fMR in patients with HF and LV systolic dysfunction was rapidly alleviated by PAP therapy. In addition, fMR revealed a stepwise reduction as the CPAP level increased, but no difference was found between CPAP at 8 cm H_2_O and ASV. Second, alleviation of fMR due to PAP was not accompanied by any overall changes in the other echocardiographic parameters, such as the SV index, fSVI, SVR index, LVEF, E/e′, and RVSP. Third, although no significant interactions were found between subgroups and alleviation of fMR, variations of the fSVI across each step were significantly affected by sex, baseline LVESV index, and baseline fSVI. fSVI increased in a subgroup of patients with a high LVESV index, whereas fSVI decreased in women and the subgroup of patients with a high baseline fSVI. Furthermore, variations of the SVR index across each step were significantly affected by the baseline fSVI. The SVR index decreased in a subgroup of patients with a low baseline fSVI. Altogether, in patients with HF, LV systolic dysfunction, and fMR, CPAP at 8 cm H_2_O and similarly ASV (EPAP of 4 cm H_2_O and automated PS ranging between 0 and 4 cm H_2_O with auto backup ventilation mode) can result in a significant alleviation of fMR, although no changes were found in the overall fSVI. Despite being results from exploratory analyses, sex, degree of LV dilatation, and degree of baseline forward SV may play some roles in hemodynamic changes that are accompanied by alleviation of fMR in response to PAP therapy.

Two studies have shown the acute effects of PAP on fMR in patients with systolic HF. In one study, Bellone et al. (2004) found that either CPAP at 10 cm H_2_O or bi-level PAP with an EPAP of 5 cm H_2_O and PS at 10 cm H_2_O for 30 min each significantly reduced the area of MR, accompanied by an increase in LVEF and a reduction in LVEDV.

The results of the present study were in line with these previous studies, although we used lower applied PAP levels over a shorter period. In addition, all patients in their study had signs of exacerbation of congestive HF, whereas all our patients were in a stable clinical state as evidenced by the absence of acute exacerbations of dyspnea. On the contrary, in another study that enrolled patients with stable HF, Haruki et al. found that a 30-min application of ASV with an EPAP of 5 cm H_2_O, PS of 3–10 cm H_2_O, and the automated backup mode did not affect the severity of MR despite significant increases in LVEF and SV and significant reductions in LVESV and SVR (Haruki et al., 2011). This lack of changes in severity of MR in their study may be explained by the fact that their study was not specifically focused on the alleviation of MR and may have included some patients without MR. Considering the findings of Bellone's study and ours, short-term application of any form of PAP (i.e., CPAP, bi-level PAP, or ASV) can alleviate fMR in patients with HF, LV systolic dysfunction, and fMR. However, acute responses of hemodynamic parameters to the PAP were inconsistent.

One mechanism for the alleviation of fMR may be that limited venous return (cardiac preload) by PAP through an increase in intrathoracic pressure leads to decongestion and reductions in LV volume and filling pressure, enhancing LVEF, and SV will occur in patients with highly dilated LV based on the Frank-Starling principle. In most previous studies on the effects of the acute application of PAP on SV, patients with high LV filling pressure and/or enlarged LV had increased SV by application of PAP (Bradley et al., 1992; De Hoyos et al., 1995; Philip-Joet et al., 1999; Steiner et al., 2008; Yamada et al., 2013). In the present study, responses of PAP on fSVI differed between subgroups, particularly subgroups divided by baseline LVESV index; in patients with a high baseline LVESV index, fSVI was increased significantly by PAP, whereas no such changes were observed in their counterparts. In addition, an increase in intrathoracic pressure by PAP can reduce the LV afterload through reduction of the LV transmural pressure and through production of a pressure gradient between the thoracic and systemic vascular system (Buda et al., 1979; Rudikoff et al., 1980; Pinsky et al., 1983). Because a failing heart is afterload-dependent, forward SV appears to be responsive to changes in afterload in patients with HF (Pinsky et al., 1983, 1985), particularly in patients at the limit of the preload reserve (i.e., close to the maximal compensatory dilation of LV) based on the Frank-Starling principle (Ross, 1976). These also explain that patients with a high LVESV index at baseline showed an increase in fSVI and that patients with a low baseline fSVI showed a decrease in the SVR index as the applied PAP level increased. By reducing both preload and afterload in addition to the increase in forward SV, PAP can improve not only dilated LV but also LV geometry (Steiner et al., 2008). Consequently, in patients with a high LVESV index, fMR can be diminished by PAP through enhanced forward SV and possibly through improved annular dilatation and increased leaflet coaptation of the mitral valve in association with the improved LV geometry (Asgar et al., 2015).

In contrast, in previous studies wherein hemodynamic responses to various PAP therapies were investigated, healthy subjects with normal or high SV and patients with HF who had relatively low LV filling pressure had reduced SV by PAP (Bradley et al., 1992; Philip-Joet et al., 1999; Steiner et al., 2008; Yoshida et al., 2012; Yamada et al., 2013). In the present study, the subgroup of patients with a high baseline fSVI had significant reduction of forward SV by PAP despite similar alleviation of MR to their counterparts. In patients with high forward SV, who may not be in an afterload-dependent state, limited preload by PAP results in a reduction of blood flow throughout the heart. This may directly cause reductions in both MR and fSVI in patients with high forward SV. Consequently, such contrasting responses of forward SV across subgroups may explain why PAP did not change fSVI despite significant alleviation of fMR in the overall patient population.

Interestingly, in the subgroup analysis of the present study, we found a significant difference in the response of fSVI to PAP between the sexes; women had reduced forward SV by PAP, whereas men did not. This may have been due to the fact that women generally have smaller LV chamber sizes and better LV systolic function than men (Redfield et al., 2005). However, because women had a similar LV chamber size and LVEF to men in the present study (see Supplementary Data), this was not the case. Because these findings are based on subgroup analysis, an exploratory analysis to generate further hypotheses, these findings should be interpreted with caution. Furthermore, the underlying mechanisms for differing responses in forward SV between the sexes may be multifactorial, and we may have to take into account the differences between the sexes in lung mechanics (Bode et al., 1976). Furthermore, lung/airway resistance and lung compliance differed based on the cardiac function (Witte et al., 2002), and it was reported that PAP improved lung compliance and lung/airway resistance in patients with HF and the work of breathing decreased as the PAP level increased (Lenique et al., 1997). Thus, investigations regarding responses of fMR and its accompanying hemodynamic parameters to PAP in consideration of lung mechanics are of interest.

A recent study suggested that adding PS favorably affects SV compared with isolated CPAP, possibly through lung inflation and reflex inhibition of sympathetic nerve activity (Yoshida et al., 2012). However, in the present study, we did not find any differences in responses of fSVI and fMR between CPAP at 8 cm H_2_O and ASV. This may be explained because much less PS than ASV was applied in the present study compared with those in the previous study (mean PS of 1 cm H_2_O in addition to 4 cm H_2_O of EPAP in the present study vs. fixed PS at 5 cm H_2_O in addition to 4 cm H_2_O of EPAP in the previous study). However, it is of great interest that ASV with an even lower net applied PAP (i.e., 5 cm H_2_O) showed similar fMR alleviation and fSVI changes to CPAP at 8 cm H_2_O (net applied PAP, 8 cm H_2_O) in the present study. This may be associated with the stabilization of respiration by ASV, which has sympathoinhibitory effects (Ushijima et al., 2014). In a study by Ushijima et al. (2014), the sympathoinhibitory effects by ASV were predominantly observed in patients with periodic breathing while awake, which is generally observed in patients with advanced HF (Tomita et al., 2015). Despite the lack of assessment of such periodic breathing while awake, the findings of Ushijima's study and our findings that patients with a low baseline fSVI who are also likely to have periodic breathing while awake showed significant reductions in the SVR index in addition to the fact that all of our patients have SDB, which frequently coexists with periodic breathing while awake (Emdin et al., 2017), may help explain why ASV showed similar alleviation of MR and changes in fSVI to CPAP at 8 cm H_2_O. Further studies assessing the effects of ASV on fMR and fSVI in consideration of the presence or absence of periodic breathing while being awake are needed to clarify this.

The present study has several limitations. First, the number of enrolled subjects was small. However, the patients' hemodynamic responses to PAP therapy in the present study are basically compatible with the results of previous studies, which indicated that PAP therapy resulted in favorable hemodynamic responses in patients who are thought to have high filling pressure (Bradley et al., 1992; Philip-Joet et al., 1999; Steiner et al., 2008; Yoshida et al., 2012; Yamada et al., 2013). In contrast, because of the small number of subjects, and because of the complex study design to evaluate the acute effect of several settings of PAP therapy on fMR and accompanying changes in hemodynamic parameters, conducting more advanced multivariate analyses is difficult, and only exploratory subgroup analyses were performed. Thus, a future study recruiting a larger number of patients in a simple comparison, for instance, between on and off PAP, is certainly required to confirm the findings. Second, the 10-min duration of each step may be too short to detect changes in echocardiography parameters. Although the duration of the application of PAP varied between 5 and 30 min in previous studies that investigated similar acute hemodynamic responses (Bradley et al., 1992; Philip-Joet et al., 1999; Haruki et al., 2011; Yoshida et al., 2012; Yamada et al., 2013), our results were similar in this perspective. In addition, it is not feasible to have a longer duration of PAP application because our protocol has five steps. Third, because the study subjects were not asleep during PAP application, extrapolation of the hemodynamic responses to PAP during sleep is difficult. Therefore, further studies are needed to determine whether the present findings can be extrapolated to patients with HF who are asleep. Fourth, although the assessment of the severity of fMR was made by previously validated methods (Helmcke et al., 1987; Spain et al., 1989; Kang et al., 2008), the color Doppler appearance of the MR jets is influenced by several parameters unrelated to MR severity, such as gain settings, packet size, aliasing velocities, and frame rate. Finally, this study was designed to assess the acute effects of PAP on the alleviation of fMR, and whether chronic application of PAP can also alleviate fMR and improve LV function remains unclear. Therefore, whether a responder in the acute phase has a similar benefit in the chronic phase needs to be determined.

In conclusion, PAP therapy, including CPAP at 8 cm H_2_O and ASV, can alleviate fMR without any overall changes in fSVI in patients with HF, LV systolic dysfunction, and fMR. However, significant differences might be found in the responses of fSVI between sexes, patients with and without dilated LV, and those with and without low baseline SV despite no such differences in the alleviation of MR. These suggest that alleviation of MR due to PAP is not always a good sign and should be interpreted with caution, particularly in patients with HF and a less dilated LV. Although the effectiveness of the long-term application of PAP for the alleviation of fMR and improvement of LV function in these patients remains unclear, patients with dilated LV who have fMR may benefit from CPAP at 8 cm H_2_O or ASV.

## Author contributions

TakaoK: Experimental design, data acquisition, and analysis, drafting of the manuscript, and final approval of the manuscript submitted. TakatoshiK and SY: Experimental design, execution, data acquisition, and analysis, drafting of the manuscript, and final approval of the manuscript submitted. AM, MH, and SM: Experimental design and analysis, drafting of the manuscript, and final approval of the manuscript submitted. HM and NS: Data analysis and drafting of the manuscript, and final approval of the manuscript submitted. MK, FK, and SS: Data analysis, revision of the manuscript, and final approval of the manuscript submitted. HD: Research financing, experimental design, data analysis, revision and final approval of the manuscript submitted.

### Conflict of interest statement

TakatoshiK, SS, HM, NS, MK, and FK are affiliated with a department endowed by Philips Respironics, ResMed, Teijin Home Healthcare, and Fukuda Denshi. HD received manuscript fees, research funds, and scholarship funds from Kirin Co., Ltd., Kaken Pharmaceutical Co., Ltd., Abbott Japan Co., Ltd., Astellas Pharma Inc., Astrazeneca K.K., Bayer Yakuhin, Ltd., Boston Scientific Japan K.K., Bristol-Myers Squibb, Daiichi Sankyo Company, MSD K.K., Pfizer Inc., Philips Respironics, Sanofi K.K., and Takeda Pharmaceutical Co. Ltd. The other authors declare that the research was conducted in the absence of any commercial or financial relationships that could be construed as a potential conflict of interest.
