# Supplementary figures and images for: Acute Effects of Positive Airway Pressure on Functional Mitral Regurgitation in Patients with Systolic Heart Failure
Source: Front Physiol. 2017 Nov 23;8:921. doi: 10.3389/fphys.2017.00921 (PMC5703848; doi:10.3389/fphys.2017.00921)

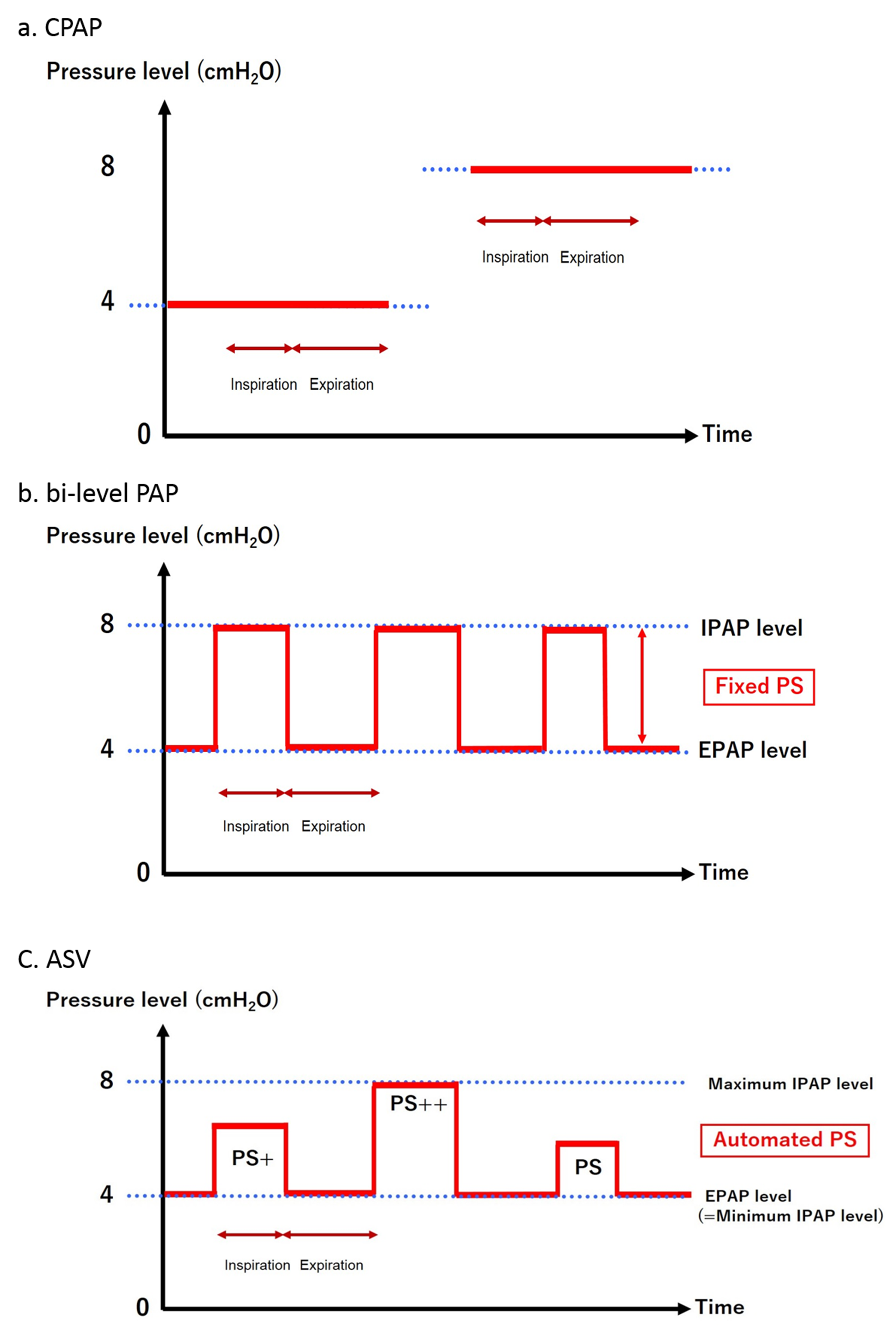

Supplement: Supplementary file 1 [file Image1.TIF]

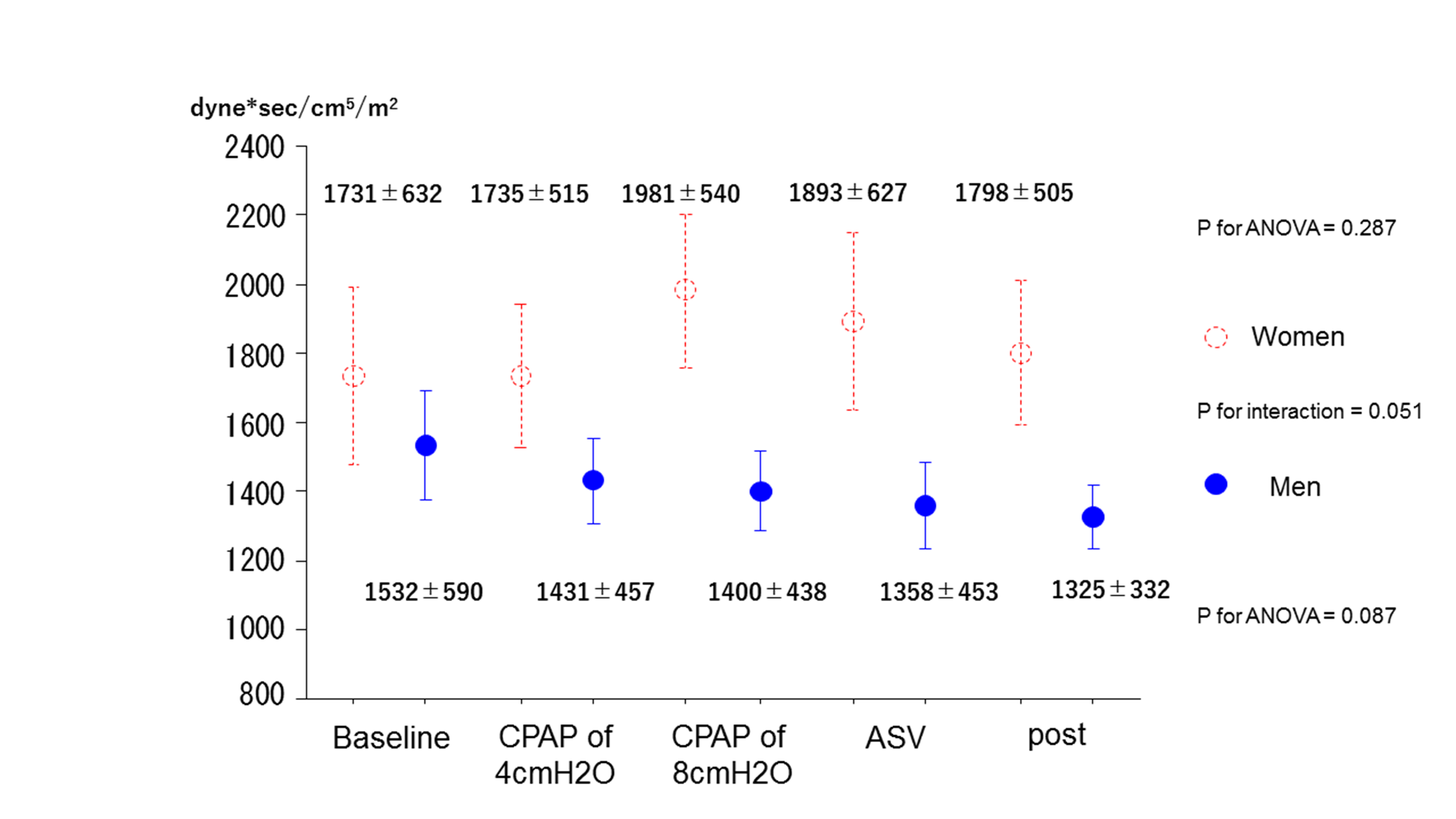

Supplement: Supplementary file 2 [file Image2.TIF]

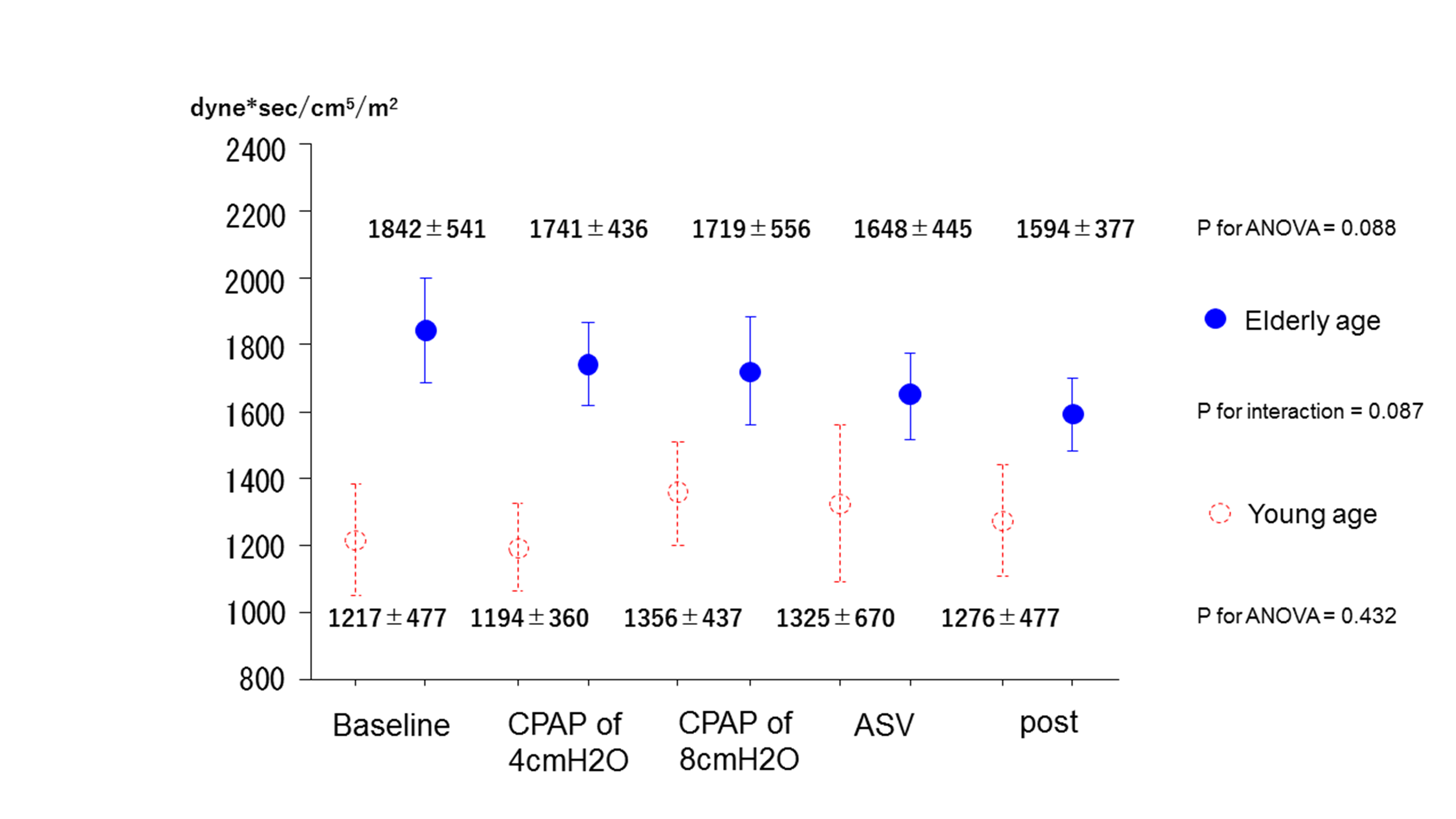

Supplement: Supplementary file 3 [file Image3.TIF]

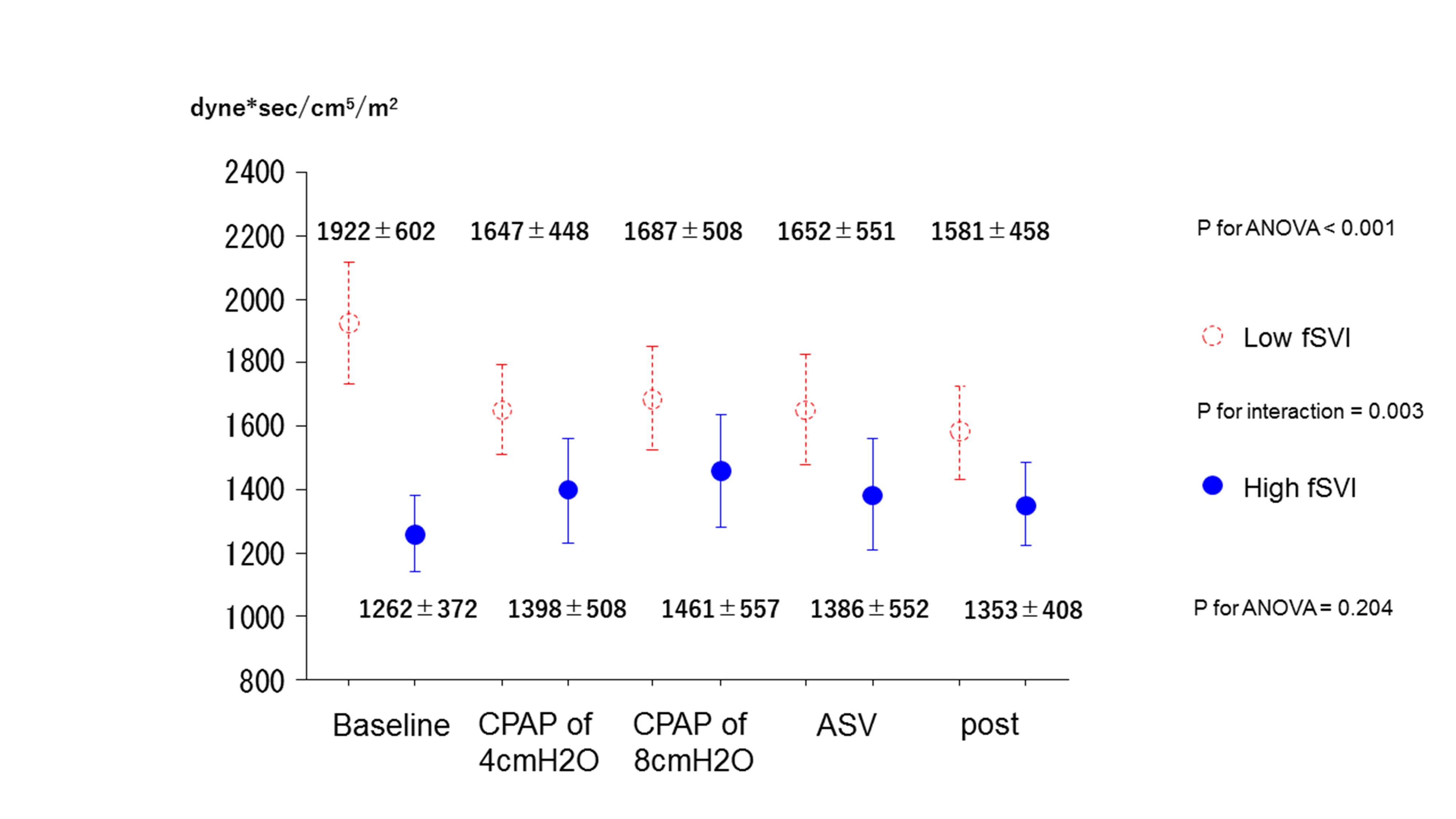

Supplement: Supplementary file 4 [file Image4.TIF]
