# Supplementary material for: Acute Effects of Positive Airway Pressure on Functional Mitral Regurgitation in Patients with Systolic Heart Failure
Source: Front Physiol. 2017 Nov 23;8:921. doi: 10.3389/fphys.2017.00921 (PMC5703848; doi:10.3389/fphys.2017.00921)
Supplement: Supplementary file 5 [file Table4.DOCX]

**Supplemental table: Baseline echocardiography parameters across sexes**

|  | Men  n=14 | Women  n=6 | P |
| --- | --- | --- | --- |
| IVST, mm | 9.8±1.8 | 9.5±2.6 | 0.776 |
| PWT, mm | 9.1±1.8 | 9.7±2.5 | 0.570 |
| LVEDV index, ml/m^2^ | 92.0±31.4 | 86.2±29.1 | 0.703 |
| LVESV index, ml/m^2^ | 61.6±27.3 | 57.1±21.3 | 0.727 |
| LVEF, % | 34.8±12.8 | 35.6±8.7 | 0.897 |
| SV index, ml/m^2^ | 30.5±9.6 | 29.1±11.1 | 0.787 |
| Forward SV index, ml/m^2^ | 26.4±10.0 | 30.0±9.2 | 0.455 |
| SVR index, dyne_*_s/cm^5^/m^2^ | 1532±590 | 1731±632 | 0.506 |
| E, m/s | 83.5 (42.0) | 78.5 (46.0) | 0.173 |
| A, m/s* | 37.0 (19.3) | 54.0 (16.8) | 0.255 |
| DcT, ms | 153.0 (87.9) | 171.5 (36.7) | 0.621 |
| e' mid, m/s | 4.0 (2.0) | 4.0 (1.0) | 0.866 |
| E/e' | 20.5 (8.4) | 19.5 (4.0) | 0.536 |
| Degree of MR, n (%) |  |  | 0.621 |
| Mild | 3 (21.4) | 2 (33.3) |  |
| Moderate | 8 (57.2) | 2 (33.3) |  |
| Severe | 3 (21.4) | 2 (33.3) |  |
| ERO area, cm^2^ | 0.33±0.08 | 0.26±0.10 | 0.178 |
| RVol, ml | 48.4±14.6 | 36.5±11.2 | 0.175 |
| MR jet area fraction | 0.36±0.12 | 0.38±0.14 | 0.734 |
| IVC, mm | 18.8±6.9 | 12.7±1.4 | 0.047 |
| RVSP, mmHg | 39.9±18.9 | 25.5±11.5 | 0.102 |

Values are expressed as mean ± standard deviation or median (interquartile range) for continuous variables and numbers (%) for nominal variables.

*n=12 in men and n=5 in women due to atrial fibrillation.

Dct, deceleration time; ERO, effective regurgitatnt orifice; IVC, inferior vena cava; IVST, intraventricular septal thickness; LVEDV, left ventricular end-diastolic volume; LVEF, left ventricular ejection fraction; LVESV, left ventricular end-systolic volume; MR, mitral regurgitation; PWT, posterior wall thickness; RVol, regurgitant volume; RVSP, right ventricular systolic pressure; SV, stroke volume; SVR, systemic vascular resistance
