# Supplementary material for: Acute Effects of Positive Airway Pressure on Functional Mitral Regurgitation in Patients with Systolic Heart Failure
Source: Front Physiol. 2017 Nov 23;8:921. doi: 10.3389/fphys.2017.00921 (PMC5703848; doi:10.3389/fphys.2017.00921)
Supplement: Supplementary file 6 [file DataSheet1.DOCX]

**Figure legend for supplemental figures:**

**Figure S1: Illustrations for differences in PAP modes**

1. **CPAP**

CPAP basically provides a constant level of positive pressure. It does not increase pressure during inspiration, and does not provide back-up ventilation.

1. **Bi-level PAP**

Bi-level PAP provides two fixed levels of PAP: a higher level of pressure during inspiration (i.e., IPAP) and a lower level of pressure during expiration (i.e., EPAP). Its major difference from CPAP is that it provides PS during inspiration and supports ventilation. In addition, most bi-level PAP devices used for patients with HF provide back-up ventilation with fixed respiratory rates but sensing spontaneous breathing.

1. **ASV**

ASV is an advanced form of bi-level PAP developed for the treatment of Cheyne–Stokes respiration in patients with HF. ASV devices automatically provide altering PS (i.e., automated PS) for each inspiration, ranging from a pre-set minimum level to a pre-set maximum level, to maintain moving target ventilation determined by the patient’s current breathing in addition to EPAP and the backup ventilation with variable respiratory rates (i.e., servo-control ventilation), leading to stabilization of respiration.

**Abbreviations:** ASV, adaptive-servo ventilation; bi-level PAP, bi-level positive airway pressure; CPAP, continuous positive airway pressure; EPAP, expiratory positive airway pressure; HF, heart failure; IPAP, inspiratory positive airway pressure; PAP, positive airway pressure; PS, pressure support

**Figure S2: Changes in the SVR index in the subgroups**

a) Men and women

The SVR index tended to vary according to sex (P for interaction = 0.051). PAP therapy tended to decrease the SVR index in men (P for ANOVA = 0.087).

b) Elderly and young patients

The SVR index tended to vary according to age (P for interaction = 0.087). PAP therapy tended to decrease the SVR index in elderly patients (P for ANOVA = 0.088).

c) High and low baseline forward SV index

There was a significant difference in variations of the SVR index according to the baseline fSVI (P for interaction = 0.003). PAP therapy decreased the SVR index in patients with a low baseline fSVI (P for ANOVA <0.001).

**Abbreviations:** ANOVA, analysis of variance; ASV, adaptive-servo ventilation; CPAP, continuous positive airway pressure; fSVI, forward stroke volume index; LVESV, left ventricular end-systolic volume; MR, mitral regurgitation; SV, stroke volume; SVR, systemic vascular resistance
